# Treatment of Upper Respiratory Tract Infections in Primary Care: A Randomized Study Using Aromatic Herbs

**DOI:** 10.1155/2011/690346

**Published:** 2010-11-01

**Authors:** Eran Ben-Arye, Nativ Dudai, Anat Eini, Moshe Torem, Elad Schiff, Yoseph Rakover

**Affiliations:** ^1^Complementary and Traditional Medicine Unit, Department of Family Medicine, Faculty of Medicine, Technion-Israel Institute of Technology, Haifa 32000, Israel; ^2^Clalit Health Services, Haifa and Western Galilee District, 6 Hashahaf Street, Haifa 35013, Israel; ^3^Department of Aromatic, Medicinal and Spice Crops, Newe Ya'ar Research Center, Agricultural Research Organization, P.O. Box 1021, Ramat Yishay 30095, Israel; ^4^The Department of Family Medicine, Ha'Emek Medical Center and the Northern District, Clalit Health Services, Afula 18101, Israel; ^5^Department of Internal Medicine, Bnai-Zion Medical Center, Haifa, 33394, Israel; ^6^The Department for Alternative/Integrative Medicine, Law and Ethics, The International Center for Health, Law and Ethics, University of Haifa, Haifa 31905, Israel; ^7^ENT Department, Ha'Emek Medical Center, Afula 18101, Israel; ^8^The Ruth and Bruce Rappaport Faculty of Medicine, Technion Israel Institute of Technology, Haifa 31096, Israel

## Abstract

This study is a prospective randomized double-blind controlled trial whose aim was to investigate the clinical effects of aromatic essential oils in patients with upper respiratory tract infections. The trial was conducted in six primary care clinics in northern Israel. A spray containing aromatic essential oils of five plants (*Eucalyptus citriodora, Eucalyptus globulus, Mentha piperita, Origanum syriacum,* and *Rosmarinus officinalis*) as applied 5 times a day for 3 days and compared with a placebo spray. The main outcome measure was patient assessment of the change in severity of the most debilitating symptom (sore throat, hoarseness or cough). Sixty patients participated in the study (26 in the study group and 34 in the control group). Intention-to-treat analysis showed that 20 minutes following the spray use, participants in the study group reported a greater improvement in symptom severity compared to participants in the placebo group (*P* = .019). There was no difference in symptom severity between the two groups after 3 days of treatment (*P* = .042). In conclusion, spray application of five aromatic plants reported in this study brings about significant and immediate improvement in symptoms of upper respiratory ailment. This effect is not significant after 3 days of treatment.

## 1. Introduction

Herbal medicine is one of the main modalities in traditional as well as complementary and alternative medicine (CAM) and is increasingly acknowledged due to the extensive use of herbal remedies amongst the general population in developed and developing countries worldwide [1, 2]. The use of herbs for treatment of respiratory ailments has been common in the Middle East for thousands of years [3]. During the last three decades, several ethnobotanical surveys were conducted in Israel and the Palestinian Authority which elucidated the use of 25 different herbs for the treatment of respiratory conditions [4, 5]. 

In vitro and clinical studies suggest the therapeutic potential of aromatic herbs in treatment of respiratory ailments. Aromatic herbs possess broad-spectrum pharmacological properties and are used as traditional remedies as well as culinary herbs. *Mentha piperita* (peppermint) contains menthol and exhibits antibacterial and antiviral properties [6] as well as an antitussive effect (in a study of guinea pigs) [7]. *Origanum syriacum* contains the active ingredients of thymol and carvacrol and possesses antimicrobial [8] and antifungal [9] properties. Boskabady and Jandaghi studied carvacrol in guinea pigs and found a bronchodilator effect [10]. A relaxant effect on tracheal smooth muscle was also demonstrated in rabbits and guinea pigs exposed to volatile oil of *Rosmarinus officinalis* (which contains camphor and cineol) [11]. Lu et al. found that *Eucalyptus globulus* oil (of which cineole is the major active ingredient) has an anti-inflammatory effect on chronic bronchitis induced by lipopolysaccharide in rats and inhibits the hypersecretion of airway mucins [12]. Kaspar et al. studied secretolytic changes of ciliary frequency and lung function in patients with obstructive lung disease and found beneficial effects of cineol [13]. Silva et al. found that both *Eucalyptus globulus* and *Eucalyptus citriodora* (containing citronellal) have central and peripheral analgesic effects as well as neutrophil-dependent and neutrophil-independent anti-inflammatory properties [14]. Cohen and Dressler conducted a clinical trial with patients suffering from acute runny nose who reported improved breathing following treatment with a volatile oil mixture of eucalyptus, menthol, and camphor [15]. In Germany, Kehrl et al. conducted a randomized controlled double-blind study in patients with acute nonpurulent rhinosinusitis and found that timely treatment with cineole is effective in reducing rhinosinusitis symptoms and is safe before antibiotics are indicated [16]. 

The limited evidence-based conventional treatment concerning upper respiratory tract infections (URTIs) [17] led the authors to study the potential of aromatic plants in alleviating URTI symptoms. Based on the evidence in the medical literature and knowledge of traditional medicine, the authors designed a prospective trial of aromatic plants growing in Israel that are often used by complementary medicine practitioners in Israel for the treatment of URTIs.

## 2. Methods

### 2.1. Study Sites and Participants

A total of 60 adult volunteers aged from 21 to 66 years were recruited to participate in this study. Participants were eligible if they were older than 18 years, had URTI symptoms and a clinical diagnosis of pharyngotonsillitis, viral laryngitis, or viral tracheitis, and gave informed consent.

Patients were excluded for a diagnosis of acute follicular tonsillitis, peritonsillar abscess, asthma, current use of antibiotic treatment, Coumadin, immunosuppressive drugs, known hypersensitivity to aromatic essential oils, and pregnancy. Primary care physicians offered eligible patients to participate in the study in six family medicine clinics in Northern Israel, operated by Clalit Health Services, the largest health maintenance organization in Israel. 

The study was administered after completing a review process and obtaining approval by the Helsinki Review Board of Ha'Emek Medical Center, Afula, Israel and registered at http://www.clinicaltrials.gov (NCT00611390). The study was performed at six primary care clinics during the winter of 2007-2008.

### 2.2. Study Design

 This study was a double-blind randomized controlled trial of a spray containing aromatic essential oils of five herbs (*Eucalyptus citriodora, Eucalyptus globulus, Mentha piperita, Origanum syriacum,* and *Rosmarinus officinalis*) compared with a placebo spray.

**Figure 1:**
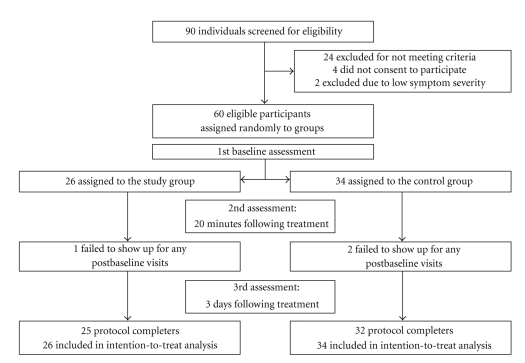
Trial profile: disposition of screened, randomized, and analyzed patients.

Figure 1 illustrates the study methodology using a three-step evaluation. After signing an informed consent form, patients were asked to participate in a baseline evaluation that included the physician's clinical assessment, a throat culture, and a record of demographics. In addition, participants were asked to assess the severity of 6 symptoms on a 4-degree scale (0: no symptoms, 1: mild, 2: moderate, and 3: severe). The symptoms assessed by the patients included the following: fatigue, sore throat, cough, hoarseness or loss of voice, pain when talking, and difficulty in breathing. Severity of the ailment (henceforth clinical score) was based on a score combining these 6 symptoms assessed by patients and 4 signs assessed by the physician (stridor, dyspnea, mucous secretion, and systemic fever >38°C). Each sign was graded as 0 (none) or 1 (positive sign). Thus, the highest severity of the clinical score of the 6 symptoms (highest score 3) and 4 signs (highest score 1) was 22. Patients were categorized as having a more severe disease if their initial clinical score was higher than 8. In order to avoid inclusion of patients with rhino-sinusitis, the severity of another 4 symptoms was assessed (including runny nose, nasal obstruction, facial and forehead pain, and difficulty when swallowing). 

Following the baseline evaluation, participants were randomized to use either a herbal or placebo spray. The sprays were packaged in identical bottles in a box containing 5 herbal and 5 placebo bottles arranged randomly. Study investigators and participants were blinded to study groups and data until the trial was completed. Participants were asked to use the spray with the indicator pointed to their throat by applying 4 sprayings each time every 5 minutes, outside the physician's room. After 20 minutes, the participants were evaluated for 3 major symptoms (sore throat, hoarseness, and cough) and general appraisal of the spray taste, smell, and other sensations. Following this evaluation, participants were advised to apply the spray at home for consecutive 3 days in a dosage of 4 sprayings each time, 5 times a day. Following 3 days of treatment, participants were invited for a final evaluation at their physician's office that included assessment of use (compliance assessment: dosage that was actually used by evaluation of empty bottles), general appraisal of the spray characteristics (e.g., sensations caused by the spray), patient's evaluation of the spray efficacy, and specific evaluation of the symptoms using the same 4-degree scale. Physicians assessed the participants' clinical status and recorded throat culture results. 

### 2.3. Aromatic Herbal Remedy Preparation

The aromatic herbal remedy was prepared in the Department of Aromatic, Medicinal and Spice Crops at the Newe Ya'ar Research Center of the Agricultural Research Organization in Israel. The fresh plant materials were hydrodistilled for 1.5 hours in a modified Clevenger apparatus. The essential oil was cooled and separated from the accompanying water. Samples consisting of 1 mL diluted essential oils (1 : 10,000 in hexane) were analyzed on a GC-MSD apparatus (6890 N, Agilent), with quadrupole detectors and electron ionization in 70 Ev, equipped with a 30 m × 0.25 mm fusedsilica capillary column (Rtx-5SIL MS, Restek). Helium was used as the carrier gas at a constant pressure of 49 psi. Injector temperature was 250°C, and transfer line temperature was 280°C. Run conditions were 50°C for 1 min, followed by temperature increase from 50° to 260°C at a rate of 5°C/min, and 6 min holding. Samples were injected in split mode. Data were collected within a mass range of 41–350 m/z. Identification of the main components was done by coinjection of authentic standards, and comparison of the electron ionization mass spectra was obtained from authentic standards and complemented with computerized libraries.

The aromatic herbal remedy was a mixture of aromatic oils of 5 herbs: 20% *Mentha piperita*, 10% *Eucalyptus citriodora*, 20% *Eucalyptus globulus*, 20% *Rosmarinus officinalis*, and 30% *Origanum syriacum*. The oil mixture was added to 97% solvent of Polysorbate 80. The final mixture was packed in 20 mL packages with a spray device. Every pressing produced fine solution drops of 0.1 mL consisting of 3 mcg of the aromatic oil mixture. Thus, patients receiving the aromatic spray were given, on the first day of the trial, a dosage of 48 mcg of the aromatic oil mixture (4 cycles of 4 sprayings each time, every 5 minutes). During the next 3 days participants used a daily dosage of 60 mcg of the aromatic mixture (4 sprayings each time, 5 times a day).

The placebo solution is constituted of water, Polysorbate 80, and authorized Lemon VIP additive (produced by Florasynth Co.) at a concentration of 0.1%. The placebo solution was packed identically to the herbal solution. The researchers assessed the placebo solution and found its taste and smell comparable to the aromatic solution although not identical. Patients' assessment of taste and smell are presented in Section 3. 

### 2.4. Statistical Analysis

Data were evaluated using the SPSS software program (version 16; SPSS Inc., Chicago, IL). The trial was designed to produce at least a 50% reduction in symptom severity between the aromatic herbal spray and control groups with Power of 80% and 95% confidence interval. The reduction was in one score on a 4-degree scale assessing three URTI symptoms (sore throat, hoarseness, or cough) comparing the aromatic herbal spray group and the control group following a 20-minute application and a 3-day followup. Pearson's chi-square test and Fisher's exact test were used to detect differences in the prevalence of categorical variables and demographic data between the participants in the two groups (herbal versus placebo spray users). The primary outcome of the study was a change in one or more of 3 URTI symptoms (sore throat, hoarseness, and cough) on a 4-score degree scale. The primary comparison was between the changes from baseline to 20 minutes in the herbal spray group compared with the control group using the chi-square tests. Paired tests, McNemar and *t*-tests, were calculated to check differences in the improvement level after 20 minutes compared to the 3-day followup in each group. *P* values less than .05 were regarded as significant. The intention-to-treat analysis included all participants for whom postbaseline values were available. In secondary analyses, the mean change in the 3-URTI symptom scores from baseline to 3 days was also compared between the herbal spray group and the control group. 

## 3. Results

### 3.1. Characteristics of Subjects

The trial profile and selection of participants are summarized in Figure 1. Among the 90 adults screened for upper respiratory tract infection, 66 met inclusion criteria. Sixty-two patients consented to participate in the study and completed baseline evaluation. Reasons for patients' refusal to participate in the study were as follows: use of other drugs or supplements for the URTI symptoms, safety concerns regarding an unproven medication, and willingness to purchase out-of-study “natural” medications with no need for doctor's supervision. Two patients consented to participate in the baseline assessment but were excluded due to low scores of symptom severity. Therefore, 60 participants were finally enrolled in the study (26 in the herbal group and 34 in the control group). Three of the 60 participants completed the evaluations at baseline and 20 minutes after applying the spray but did not use the spray for the next 3 days as instructed by the study protocol. 57 of the 60 participants used the spray as instructed (compliance rate: 95%). Nevertheless, intention-to-treat analysis was performed for all 60 participants. 

The two study groups had comparable demographic and clinical characteristics including age, gender, urban/rural residence, days of illness prior to inclusion, fever, recurrent tonsillitis background, and severity of debilitating symptoms (Table 1). In the control group, more participants reported current smoking (29% versus 8%), but this difference was not significant (*P* = .052). Throat cultures were positive for two patients from the group receiving the herbal spray and one patient in the placebo group (all of them eventually received antibiotic treatment).

### 3.2. Clinical Outcomes

In the intention-to-treat analysis, there was a significant difference between the clinical scores of the treatment and control groups 20 minutes following the spray use (Table 2). Participants in the study group reported more improvement in the severity of their most debilitating URTI symptoms compared to participants in the placebo group (*P* = .019). Improvement was defined as reduction of at least one score in symptoms of sore throat, hoarseness, or cough. Analysis of a sub-population of 46 patients with the more severe clinical score (the clinical score is described in Section 2 and combines 6 symptoms and 4 signs) also showed a significant difference between the treatment and placebo groups (*P* = .009).

There was no difference in symptom severity between the two groups after 3 days of treatment (*P* = .42) (Table 2). 

Adverse effects due to therapy in both groups were similar and mild in nature, such as a dry pharynx and/or a slight stinging sensation. Assessment of blinding was based on the participants' report of the spray taste, smell, and burning and dry sensation following application. Participants in both groups graded overall spray efficacy similarly. Intermediate to good efficacy was reported by 16/26 participants (61.5%) in the aromatic spray group and by 18/33 (54.5%) in the placebo group. Compared to the placebo group, fewer participants in the aromatic group attributed moderate to good taste to the spray (9/25 (36%) versus 8/17 (47%)) but reported a better smell (19/25 (76%) versus 7/18 (38.8%)).

Assessment of compliance was based on the participants' reports. Participants in both groups reported taking maximal dose of spray in the first day of treatment (26/26 participants in the aromatic spray group versus 33/34 in the placebo group). In day 3 of treatment, 25/26 participants in the aromatic group reported compliance with spray use (18 reported maximal recommended dosage and 7 partial dosage) compared to 32 participants in the control group (25 reported maximal dosage and 7 partial dosage).

## 4. Discussion

In this randomized double-blind trial, the effect of a herbal preparation on URTI symptoms was studied compared to placebo. Patients reported a significant decrease in URTI symptoms 20 minutes postadministration (*P* = .019). Analysis of a sub-population of patients with severe symptoms (46 participants) showed an even more significant difference between the treatment and placebo groups (*P* = .009).

**Figure 2:**
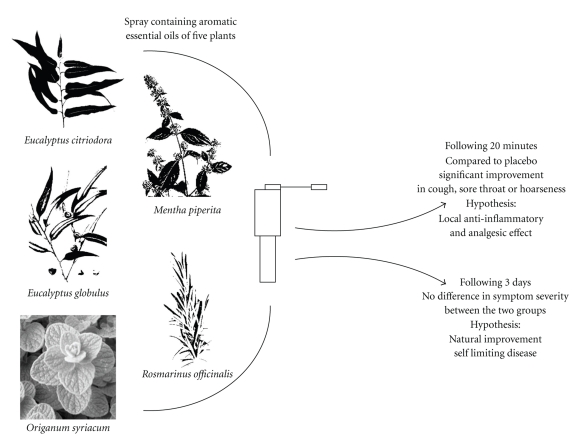


As can be expected in upper respiratory tract infections, participants in both groups significantly improved after 3 days. No statistical difference was detected between the herbal and placebo groups after 3 days. These findings suggest that the studied herbal preparation possesses a local, rather than systemic, effect on the upper respiratory tract. Following 3 days of treatment, this beneficial effect was minimized, possibly due to natural improvement of patients' symptoms that typically characterize URTI (Figure 2).

The trial was conducted in community family medicine clinics and, therefore, reflects URTI management in primary care rather than the more symptomatically severe population referred to ENT clinics and hospital departments.

This study has several limitations. The small sample size may limit the interpretation of the study results; hence, further research is warranted with a larger number of participants. In addition, although participants in the two groups had comparable characteristics, smoking in the control group was more prevalent and nearly significant (*P* = .052), thus possible influence of smoking on participants' taste and smell cannot be excluded. Another limitation is the use of only three points of clinical evaluation. Continuous measures of symptoms (e.g., patient's symptom diary) may contribute to learning about the clinical efficacy of the plant spray along the three days of evaluation. Another limitation is concerned with the theme of herbal safety. Although we did not observe any severe side effect symptoms, larger-scale studies are warranted in order to conclude the safety of the aromatic herbal formula. An additional limitation of our study relates to the challenging issue of blinding in herbal studies, especially when dealing with aromatic herbs. Although we did not assess participants' blindness, we did receive the participants' assessment regarding the smell and taste and their overall opinion regarding the spray's efficacy. Interestingly, participants receiving the aromatic spray reported a better smell compared to the placebo group, but fewer participants reported a good taste. In view of the fact that the aroma of aromatic herbs includes both smell and taste, we cannot conclude that blinding in our study was disrupted. Nevertheless, we recommend direct assessment of blinding in further clinical studies using aromatic herbs.

In order to clarify our findings, we reviewed the literature and found three randomized controlled studies that studied remedies containing aromatic herbs in a clinical setting of URTIs. Hubbert et al. studied the efficacy and tolerability of a spray with *Salvia officinalis* (containing camphor) in the treatment of acute pharyngitis [18]. The researchers reported that pain relief occurred within the first two hours after the first administration. In another double-blind randomized controlled herbal trial, Dirjomuljono et al. examined the effect of two herbs, *Nigella sativa* and *Phyllanthus niruri*, in patients with acute tonsillopharyngitis symptoms. In this study, the alleviation of swallowing pain and difficulty was noticed only after 5-6 hours, presumably due to a systemic effect (oral tablets versus spray application in other studies) [19]. Design of herbal studies in the URTI setting needs to take into account the various herbal constituents and activities in order to choose the most appropriate route of administration. For example, *Nigella sativa*, a well-known herbal remedy in Islamic medicine, possesses an antispasmodic effect and can increase mucociliary clearance; thus, a systemic rather than local activity may be expected [20]. A third option for treating sore throat and other URTI symptoms is with a herbal tea preparation that may act both locally and systemically. Brinckmann et al. demonstrated significant relief of pain in patients with acute pharyngitis within 5 minutes after drinking *Throat Coat*, a traditional demulcent herbal tea, compared with placebo [21]. Other promising remedies are the traditional Japanese (Kampo medicine) formulas named Hochuekkito (TJ-41) and Juzentaihoto (TJ-48) that stimulate the mucosal immune system of upper respiratory tract [22]. In our study, we designed application of aromatic oils in spray form rather than tea or tablets. We intended to gain the local benefit of these evaporating aromatic oils, which may attain better penetration of the tonsils' folders and other pharyngeal structures.

The rapid symptom improvement 20 minutes following application of the herbal spray may be explained by an anti-inflammatory and analgesic effect. Indeed, human and animal studies concluded that eucalyptus [14] and mint oil [6] possess these effects. The antitussive effect of menthol and camphor [7] and the bronchodilating effect of carvacrol [10] may also explain the rapid symptomatic relief observed in our study. However, this study cannot conclusively determine that the combination of the 5 herbs is indeed essential. Further clinical studies are warranted to determine the dosage of different herbs in the formula for relieving URTI symptoms and whether these herbs possess cumulative or synergistic effect.

The advantages of the aromatic preparation reported in our study may not be limited to patients' relief due to symptom improvement. Plant extracts are potential sources of antimicrobial and resistance-modifying agents and may be used to decrease antibiotic resistance [23]. Moreover, utilization of complementary and alternative medicine (CAM) therapies, including herbal preparations, could reduce prescribing antibiotics in cases when there is no clinical indication, such as viral infections [24].

## Figures and Tables

**Table 1:** Demographic characteristics of the randomized patient population (*n* = 60).

	Overall (*n* = 60)	Herbal group (*n* = 26)	Control group (*n* = 34)	*P* value
Age, years, mean (SD)	43.9 ± 12.4	46.6 ± 11.9	41.9 ± 12.6	0.19^a^
Sex (%)				
Female	32 (53%)	13 (50%)	19 (56%)	0.79^b^
Male	28 (47%)	13 (50%)	15 (44%)	
Residence, *n*				
Urban	42 (70%)	19 (73%)	23 (68%)	0.78^b^
Rural	18 (30%)	7 (27%)	11 (32%)	
Days of illness prior to inclusion, *n*				
One day	10 (17%)	4 (15%)	6 (17%)	0.96^b^
Two days	22 (37%)	10 (39%)	12 (35%)	
More than two days	28 (47%)	12 (46%)	16 (47%)	
Fever >37.5°C prior to inclusion, %				
Yes	14 (23%)	6 (23%)	8 (24%)	1.00^b^
No	46 (77%)	20 (77%)	26 (76%)	
Recurrent tonsillitis, %				
Yes	4 (7%)	3 (12%)	1 (3%)	0.31^b^
No	56 (93%)	23 (88%)	33 (97%)	
Current smoker, %				
Yes	12 (20%)	2 (8%)	10 (29%)	0.052^b^
No	48 (80%)	24 (92%)	24 (71%)	
Baseline assessment of the most debilitating symptom^c^				
Cough/Hoarseness mean (SD)				
Score 1	7 (12%)	3 (11%)	4 (12%)	0.35^b^
Score 2	29 (48%)	10 (39%)	19 (56%)	
Score 3	24 (40%)	13 (50%)	11 (32%)	

^a^ *t*-test ^b^Fisher's exact test ^c^The most severe of the following symptoms: sore throat, hoarseness, or cough.

**Table 2:** Changes in clinical scores of the most debilitating symptoms from baseline to 20 minutes following spray application and 3-day followup.

	Overall (*n* = 60)	Herbal group (*n* = 26)	Control group (*n* = 35)	*P* value
Clinical improvement* in the most debilitating symptom 20 minutes following application, *n* (%)	33 (55.0)	19 (73.1)	14 (41.2)	.019
Clinical improvement* in the most debilitating symptom following 3 day application, *n* (%)	38 (63.3)	18 (72.0)	20 (58.8)	.42

*Improvement was defined as at least one-score reduction in a 4-degree symptom scale ranging from 0 to 3 of the most debilitating of the following symptoms: sore throat, hoarseness, or cough.
